# Biliary tract injury after cholecystectomy: a surgeon’s worst nightmare

**DOI:** 10.1097/JS9.0000000000000474

**Published:** 2023-05-20

**Authors:** Jianwei Li, Dingwei Xu, Jie Huang

**Affiliations:** a Department of Hepatobiliary Surgery, Southwest Hospital, Army Medical University, Chongqing; b Department of Hepatopancreatobiliary Surgery, The Second Affiliated Hospital of Kunming Medical University, Kunming, Yunnan, People’s Republic of China

*Dear Editor,*

Cholecystectomy is a commonly performed surgical procedure with an estimated annual volume of 750 000 in the USA^1^. Despite its low incidence, which ranges from 0.1 to 2.3%, bile duct injury (BDI) remains a severe complication that can result in significant morbidity, mortality and healthcare costs^2^. Furthermore, BDI often leads to high rates of medicolegal claims. Despite the implementation of a critical view of safety introduced by Strasberg, recent studies and recommendations regarding the management of biliary disease have not significantly reduced the BDI incidence. Many patients who experience BDI have a poor quality of life and financial burden, while subsequent litigation can create liability for hospitals and surgeons involved in their care. Given the complexity of BDI following cholecystectomy, it is imperative for surgeons, gastroenterologists and radiologists to work together to address it^3^.

The absence of appropriate surgical equipment compromises cholecystectomy outcomes because each unsuccessful attempt results in further reduction of bile duct length and can escalate BDI from E2 to E3 or E4^4^ while creating challenges for subsequent repair attempts and requiring liver resection in some cases. Therefore, adopting a drain-now-and-fix-later approach is deemed safe, emphasising the importance of early referral to specialised centres. We congratulate the authors on their comprehensive work on assessing the impact of reconstruction time and abdominal sepsis control on the rate of reconstruction success based on a multicentre randomised controlled trial ^4^.

Mohammed *et al.* concluded that early reconstruction after abdominal sepsis control could be safely performed at any time, with outcomes comparable to those observed with delayed reconstruction, in addition to decreased total cost and improved health-related quality of life. However, we have certain reservations regarding the study.

First, this was a multicentre, multiarm, parallel group, randomized controlled trial that included all consecutive patients treated with hepaticojejunostomy for major post-cholecystectomy BDI from February 2014 to January 2022. However, we do not consider this to be a randomised controlled trial, in which participants are randomly assigned to a treatment or control group, and only the treatment group receives the study intervention. Randomised controlled trials include several key elements such as randomisation, a control group, blinding and outcome measures. In addition, patients are randomly assigned to either the treatment or the control group to ensure that the groups are similar in all aspects except for treatment, and the trial is conducted in a blinded manner where the patients, researchers and data analysts are unaware of the group assignments of the patients. However, in this study, the patients were allocated to specific groups based on their biliary status rather than random allocation. However, we understand that allocation to specific groups would be both unethical and impractical in this study.

Second, the patients were followed biweekly during the first 3 months and monthly thereafter during the first year, and annual follow-up evaluations were conducted after the first year. As shown in Table 3 of the study, the longest follow-up was 41 months. Conversely, most patients with symptomatic biliary stricture after bile-intestinal anastomosis develop symptoms 5 years or later after surgery. Some studies reported that the median time to biliary-enteric anastomotic strictures ranged from 11 to 30 months, with ~66, 80 and 90% of the cases occurring within 3, 5 and 7 years, respectively. The long-term mortality rate following treatment for BDI ranges from 1.8 to 4.6% and failure to recognise or treat strictures can lead to secondary biliary cirrhosis, associated complications and death^3^. Therefore, we contend that the follow-up period was inadequate in this study by Mohammed *et al*.

Finally, while we recognise the high utility of the administrative databases in this multicentre study, the heterogeneity in data collection and the variations in the skill level of surgeons might have significantly impacted the study findings. Additionally, the experience level of individual surgeons might have differed across the cases.

In conclusion, we express our gratitude to the authors for their findings. Nevertheless, further research is imperative to adequately assess the impact of treatment on biliary tract injury following cholecystectomy.

## Ethical approval

This paper is a correspondence and does not require ethical approval.

## Consent

This paper is a correspondence and does not require consent.

## Sources of funding

This work was supported by the Yunnan Fundamental Research Projects (Grant No. 202101AT070239) and the Investigator-Initiated Trail Projects of the Second Affiliated Hospital of Kunming Medical University (Grant No. 2020ynlc004).

## Author contribution

J.L.: conceptualisation, data curation, formal analysis, investigation, and methodology; D.X.: writing – original draft preparation; J.H.: data curation, supervision, and writing – reviewing and editing.

## Conflicts of interest disclosure

No potential financial or non-financial conflicts of interest.

## Research registration unique identifying number (UIN)

Not applicable.

## Guarantor

Jie Huang.

## Provenance and peer review

Not applicable.

## Data availability statement

Not applicable.
